# Tailoring Our Approach in Response to the SARS-CoV-2 Pandemic and Transcending Science Outreach Modalities for Native American Students in a Cancer Research Education Program

**DOI:** 10.15695/jstem/v4i4.08

**Published:** 2021-10-04

**Authors:** Aislinn C. Rookwood, Liliana P. Bronner, Mariah A. Abney, Hannah S. Butler-Robbins, Misty S. Pocwierz-Gaines, Alaina C. Larson, Alexis M. Huckleby, Joyce C. Solheim, Maurice Godfrey, Regina E. Idoate

**Affiliations:** 1 Department of Health Promotion, University of Nebraska Medical Center, Omaha, NE; 2 Department of Family Medicine, University of Nebraska Medical Center, Omaha, NE; 3 Eppley Institute and the Fred and Pamela Buffett Cancer Center, University of Nebraska Medical Center, Omaha, NE; 4 Munroe-Meyer Institute, University of Nebraska Medical Center, Omaha, NE

**Keywords:** Cancer, high school, middle school, Native American, American Indian, Alaska Native, online, undergraduate

## Abstract

In response to the SARS-CoV-2 pandemic, a cancer research education program at the University of Nebraska Medical Center designed for Native American middle school, high school and undergraduate students adapted activities to be delivered online. There are considerable challenges to adapting in-person science programming to online delivery that can impact overall effectiveness. These challenges are further exacerbated when the cognate student population also faces significant disparities in health, wealth, and educational outcomes. We encountered both disadvantages and advantages in transitioning programming to online virtual formats. Challenges faced in delivering our programming during the pandemic included varied online accessibility, peripheral stressors, and disconnection to places and people. Despite these challenges, we found several benefits in remote delivery, some of which have alleviated barriers to program participation for Native American students. Some successes achieved by transitioning to fully remote programming included increased program reach, sustainability, and cultural relevancy. In this overview of the implementation of four online programs at the middle school, high school, and undergraduate levels, we highlight the challenges and successes experienced. Through this program description, we aim to provide insight into potential strategies to improve program delivery designed for Native American students during the SARS-CoV-2 pandemic and beyond.

## INTRODUCTION

### Impact of the SARS-CoV-2 Pandemic on Native American Education.

The National Indian Education Association maintains that the SARS-CoV-2 pandemic overturned science education programming and delivery to Native American (NA) students (NIEA, 2020). Approximately 22% of NA students lack access to the internet in their homes (NCES, 2020). Furthermore, many NA students live in multigenerational homes and may not have dedicated space for formal learning (USCCR, 2018). NA students are generally considered “field sensitive” learners, meaning that they prefer to work with others, observe real-life demonstrations and modeling, and deeply value personal relationships with their teachers and peers (Cajete, 1999). All the essential components known to enhance NA students’ motivation and engagement with respect to education and science, including the importance of relationships, culture, hands-on experiential, and collaborative learning (Herrington, 2014; Kant et al, 2018), were challenged by a virtual and remote environment.

### The UNMC Ignite-Engage-Sustain YES Program.

**Figure 1.**
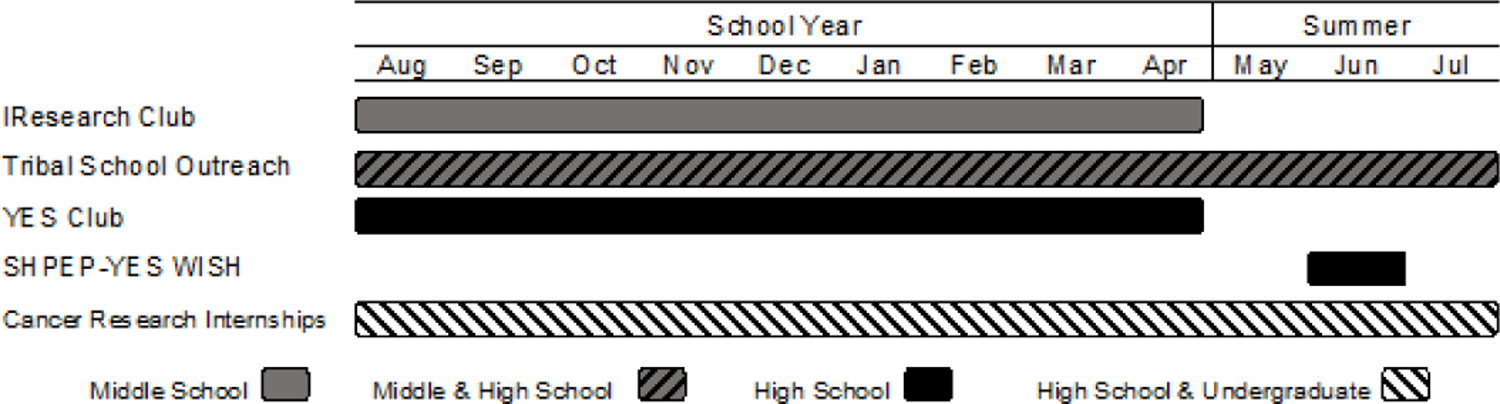
Overview of UNMC YES Program delivery by grade level and time of year

In the United States (US), American Indian/Alaska Native (AI/AN) people have the worst survival rates for virtually every cancer type (NCI, 2018). Having more cancer healthcare workers and scientists with the perspective of the AI/AN people would be advantageous in remedying this disparity, but very few AI/AN people are entering cancer-related careers and their rate of matriculation into medical and science/engineering graduate or professional programs is extremely low (AAMC, 2019; NSF 2021). In response to this need, the University of Nebraska Medical Center (UNMC) developed the Ignite-Engage-Sustain or Youth Enjoy Science (YES) pathways program, funded by a National Cancer Institute’s Youth Enjoy Science (YES) Research Education (i.e., R25) grant. The UNMC YES program aims to motivate, involve, educate, and mentor NA students and their communities in cancer prevention, treatment, and research. The long-term goals of this program are to encourage, support, and prepare NA students for biomedical or public health careers in cancer research through programming and outreach at the middle school, high school, and undergraduate levels (Figure 1). University faculty and staff collaborate with biomedical scientists, school teachers, community members, and students to develop and sustain innovative research experiences, curriculum, and outreach strategies that attract and engage NA students and their teachers in active cancer research environments. The UNMC YES program also aims to provide culturally appropriate experiences and curriculum that acknowledge students’ diverse backgrounds, knowledge, interests, strengths, and challenges. Student engagement in the UNMC YES program is enhanced through summer experiences such as science camps for middle school students, enrichment programs for high school students, and long-term research internships with the support of mentors for high school and undergraduate students.

Below, we describe our adaptations in programming due to the SARS-CoV-2 pandemic, as well as the advantages and disadvantages experienced in transitioning to online delivery. We share our story to highlight considerations for students, educators, and evaluators/researchers supporting NA students in cancer research education and other types of STEM programs. We share the evaluation of four programs (the middle school IResearch club, high school YES club, SHPEP-YES WISH program, and YES Cancer Research Internship) and the lessons learned which are applicable to program delivery during and after the end of the SARS-CoV-2 pandemic’s restrictions.

## PROGRAM ADAPTATIONS

### Middle School Outreach and Indigenous Research (IResearch) Club.

Before the start of the SARS-CoV-2 pandemic, tribal school outreach in Nebraska and South Dakota communities was offered in person by the UNMC YES program on several occasions as day-long events. Since the inception of UNMC YES in 2017, program scientists have regularly visited tribal schools where they engaged students in hands-on cancer-related science education. For example, in 2018, YES scientists visited the Omaha Nation middle school to teach students how to extract DNA. We also invited tribal school students in the region to attend a oneday workshop, *Cancer Biology and You Day*, that was hosted by YES personnel and graduate student mentors every other year (Herek et al., 2019). However, because the pandemic caused all regional tribal lands to be closed to visitors, YES tribal outreach has been paused. With many public school districts’ instruction held online, access to local university campuses restricted, and regional tribal reservations closed in response to the SARS-CoV-2 pandemic, we worked creatively and diligently to modify program delivery to transition programming to online platforms in 2020.

In the Omaha metro area, YES personnel facilitate a school-based Indigenous research (IResearch) club focused on topics of interest to the community. The IResearch club program is offered through the UNMC YES program to NA middle school students interested in health science professions. This club is active year-round, investigating cancer-related health concerns identified through a Community Readiness Assessment (Idoate et al., 2020) with input from teachers and students in the public school district’s Native Indigenous Centered Education (NICE) program. Prior to the pandemic, YES middle school IResearch club programming was conducted in three middle schools as part of the NICE program. IResearch programming was delivered during school hours through either in-person meetings at lunch and study hall at each individual school or quarterly field trips with all participating schools on a university campus or in the community. Participants attended field trips to places that were relevant to each specific research project. In early 2020, students in the IResearch club visited regional rivers to measure nitrates in surface waters for a citizen science project.

Due to the spread of SARS-CoV-2, on March 1, 2020, the public-school district began delivering adapted programs through the district’s preferred online communication tool, Microsoft Teams. YES IResearch responded by acquiring Microsoft Teams to transition IResearch program delivery to this virtual platform. YES personnel collaborated extensively with the NICE teachers to develop the technological skills necessary for students, teachers, and external stakeholders to work with YES through the virtual platforms available. Making changes to centralize resources to respond to the shifts from in-person learning, to fully remote learning, to hybrid formats for the IResearch clubs required flexibility and a comprehensive approach to our adaptations. The time dedicated to preparing adaptations to programming resulted in a two-month pause in extracurricular program delivery.

At the start of the new school year in Fall 2020, students began a new project assessing residential radon in their homes. Participation involved 100% online meetings with fellow students, NICE teachers, YES personnel, and representatives from community and local organizations, such as the Omaha Healthy Kids Alliance (OHKA). In November of 2020, the NICE program, in accordance with the public school district’s policies, transitioned to a 3/2 instructional mode restricting in-person class size gatherings by student last name. Half of the student population attended school in-person Monday and Tuesday, and the other half of the student population attended school in-person each Thursday and Friday. Wednesday school sessions alternated between the two halves of the student population. Students and their families also had the option to elect to continue fully remote instruction.

New approaches were devised to meet the varying student schedules. IResearch in-person meetings and field trips were disrupted by the SARS-CoV-2 pandemic. In-person meetings were adapted to online meetings using Microsoft Teams. Instead of holding individual meetings at each school, we held one virtual meeting for all NICE students across all middle schools. Online meetings were held during the students’ lunchtimes, sometimes offered in duplicate or triplicate to accommodate all participating students. In addition to live meetings, e-learning modules and practical educational materials developed by YES personnel were made available to students and teachers through Microsoft Teams. Some educational materials were provided to students in hard copy via personal delivery or distribution through schools. In addition, educational materials were made available through Microsoft Teams as fillable forms that could be completed online. In place of field trips, we invited guest speakers to share information via virtual presentations about community-based organizations and projects, hoping that students will be able to visit these places and people in person in the future.

### Summer Health Professions Education Program (SHPEP) Weeklong Institute for Students in High School (WISH) Program.

The WISH summer enrichment program is offered through the UNMC YES program to NA students interested in health science professions. This program is a part of the larger, national SHPEP for undergraduate students funded by the Robert Wood Johnson Foundation. The program aims to strengthen the career development of students underrepresented in the health professions and improve students’ access to information and resources that can prepare them for college success in a health-science field. WISH provides additional culturally relevant sessions on cancer research, prevention, and health care.

In the summers before the SARS-CoV-2 pandemic, WISH participants attended onsite programming and lived in student residence (i.e., dormitory) halls alongside SHPEP undergraduates from across the nation. In response to the SARS-CoV-2 pandemic, all SHPEP programming was moved from in-person, on-campus events to 100% online meetings and activities. All originally planned visits to campus resources, cancer research areas and the clinical simulation laboratory were cancelled. As college campuses began to close, so did student residence halls, eliminating the opportunity for WISH students to live among other program participants. Peer-to-peer learning opportunities were reduced.

In accordance with SHPEP, the WISH program announced its transition to online delivery via Canvas in April 2020. Our remote curriculum included synchronous Zoom video conferencing, pre-recorded asynchronous sessions, and discussion boards to engage the WISH students in learning from each other and encourage relationship building. YES personnel communicated regularly with WISH participants through email, Canvas, and messaging through a mobile application, GroupMe. GroupMe can sync with Apple or Android phones and protects student privacy by not showing their phone number externally.

The most difficult components to orchestrate virtually were the informal interactions and casual information exchanges that had commonly happened between WISH participants and SHPEP undergraduate students and faculty. In place of regularly scheduled social events, the WISH program organized talking circles specifically covering various dimensions of wellness, college preparation, and health profession and research career development. The WISH students were paired with graduate/professional student mentors in the online platform with whom they met in a more formal talking circle for a scheduled hour each day.

### Undergraduate and High School Student Cancer Research Internships.

Through the UNMC YES program, we offer undergraduate students and high school students (≥16 years old) with NA ancestry opportunities to participate in community-based cancer research projects at the College of Public Health and/or biomedical lab-based research projects in the Fred and Pamela Buffett Cancer Center. Interns typically work with graduate students, research personnel, and a faculty mentor on these projects. UNMC YES program students who live outside of the Omaha metropolitan area (where our campus is located) have historically been invited to participate in summer research projects and been provided financial support for housing, food, and travel to Omaha. Students located in the Omaha metropolitan area have regularly participated in part-time projects during the school year and/or over the summer. As much as possible, research experiences are tailored to students’ interests and availability. Some examples of research projects that interns have participated in include: investigating an Osage elder’s experience of living with skin cancer (McWilliams and Idoate, 2020), investigating the association between the quantity of a specific enzyme and the presence of a protein on melanoma cells that allows recognition by immune cells, analyzing proteins that contribute to the growth and spread of pancreatic cancer, conducting a community readiness assessment to develop stage-appropriate strategies to address cancer in the community, and examining the functional role of genes mutated in breast cancer tumors in chemotherapy resistance (Idoate et al., 2020).

On April 8, 2020, the UNMC campus was closed to all individuals except essential personnel, in-person research was restricted, and all work with cancer laboratory research interns was postponed for the remainder of the spring and the summer semesters. In-person internships in the Eppley Institute laboratories in the Fred and Pamela Buffett Cancer Center planned for the Fall 2020 and Spring 2021 semester were redesigned as online internships. One laboratory intern, who had earlier begun in-person, was invited to department seminars and weekly lab meetings with her mentor to continue her cancer research training remotely. Additionally, she assisted a postdoctoral fellow in the lab with editing a scientific manuscript that was submitted to an academic journal for peer review.

An online laboratory-oriented YES internship within the Fred and Pamela Buffett Cancer Center was developed by YES personnel and a Cancer Research Doctoral Program student mentor. This internship utilizes a Canvas course to deliver laboratory curriculum. This course is designed to give the interns a richer appreciation of the experience of a laboratory-based cancer research project. The course introduces laboratory interns to a variety of research techniques that are commonly used in biomedical laboratories as well as more personalized laboratory tour videos. In addition to the interns engaging with each module on their own time, the course plan includes regular meetings of the interns with the graduate student mentor. These meetings are intended to provide time for the interns and student mentor to discuss the weekly module as well as what the graduate student mentor is doing in the laboratory that week. Through these meetings, the intern can apply the newly acquired knowledge of research techniques to the research being conducted by the student mentor. Currently, one YES intern is on track to complete this Canvas-based course, another has recently enrolled, and a third has expressed interest in undertaking it in Fall of 2021.

Research projects involving interns in the College of Public Health were minimally disrupted by the pandemic. All research continued and modifications to study protocol switched from in-person to online delivery. For example, in place of conducting in-person interviews, interns conducted interviews via Zoom, and in place of presenting research in-person at conferences, interns presented research in virtual conferences via online platforms.

## LESSONS LEARNED

Internal formative and process evaluation of our programming from January 2019 to July 2021 has identified both challenges and successes to delivering online UNMC YES programming during the pandemic. Program evaluation results were derived from the analysis of student surveys, student engagement via online chat, student interviews, and advisory board listening sessions. The Canvas-based YES internship program that we are now piloting will be evaluated later after more students enroll and complete the course. As described in detail below, the challenges we faced included varied online accessibility, peripheral stressors, and disconnection to places and people. The successes we experienced included improvements in program reach, sustainability, and cultural relevancy.

### Challenges.

#### Varied Online Accessibility.

YES personnel and partners from community-based organizations had varied and mismatched online teaching/learning readiness levels and technologies available to them (e.g., Zoom vs. Microsoft Teams, a range of internet providers, Canvas vs. e-learning modules). Middle and high school students worked to adapt rapidly to using email and video conferencing programs as primary modes of learning and communication, in some cases for the first time. This adjustment was particularly challenging because online platforms required greater cognitive effort to navigate. The necessity for these adjustments delayed some programming. It took extra time for our personnel, partners, and students to learn new software and its use. Undergraduate students were proficient in using email and video conferencing tools, like Microsoft Teams and Zoom. However, YES interns reported to YES personnel that they experienced difficulties accessing these platforms during regular working hours because of limited internet access, low broadband width at home, life distractions (e.g., family concerns, working at additional jobs), or other technological difficulties (e.g., a UNMC system-wide cyber-attack, Out-look malfunctions).

Many students from the population we serve do not have regular access to personal smartphones, computers, or tablets. Those who do often have inadequate internet access to support online learning platforms and video conferencing (Table 1). Therefore, online access was not available to all possible participants, potentially limiting UNMC YES student enrollment and participation. As previously discussed, some school districts provided devices to their students for online learning. However, it was reported to YES personnel that some middle and high school students did not receive their school district-issued devices by anticipated deadlines due to outdated contact information. In addition, some students with devices lived in urban areas where there were “internet dead zones” (Siegler, 2020) and could not access the internet to attend YES meetings with consistency. Moreover, partner schools and programs in rural communities were faced with limited accessibility to installing internet connections despite having district-provided funds to do so. As a public health precaution to reduce risk to employees and customers, small internet providers restricted their employees from accessing customers’ homes for installation. This situation created an added barrier for students.

IResearch club attendance varied greatly throughout all meetings in comparison to the previous year of programming. Participation was influenced by online accessibility with many students unable to access reliable, wireless internet through devices. During the schoolyear pre-pandemic, 28% of participants attended all program sessions compared to less than 1% who attended all virtual program sessions. All student participants (100%) received program materials distributed through schools or personal home delivery. However, this required additional coordination on the part of the YES personnel and partner teachers.

The WISH on-boarding process required extensive self-motivation on the part of the students and additional follow-up from YES personnel to complete. Historically, YES personnel have met in person and worked closely with interested NA students to review the program onboarding process. This process includes completing a college application for admission to UNMC, downloading apps on devices to authenticate access to the university network, and accessing various systems to update the student’s information. Some of these steps require access to a computer and cannot be completed on a mobile device (e.g., tablet, cellular phone). Some students reported only having access to mobile devices, excluding some students from participation. For WISH programming, we utilized Canvas to house all program materials and curriculum. However, accessing Canvas requires students to complete multiple security procedures (e.g., using an app on a mobile device or phone for identity authentication and entering login credentials), which, at times, hindered participation.

#### Peripheral Stressors.

The new online learning environment proved to be both mentally and emotionally overwhelming for students, school partner teachers, and YES personnel (Table 1). Many YES participants did not have their own private space at home to dedicate to school and, understandably, many students were not comfortable bringing a camera into their home environments. We understood that many students could be “self-conscious about being seen in class, weren’t in private spaces and/or didn’t want to show their current living situations” (Nicandro et al., 2020). To accommodate for this, YES IResearch club teachers, WISH chaperones and YES internship mentors did not require students to have their cameras on during meetings.

UNMC YES personnel working off-campus also had additional responsibilities at home (e.g., caring for young children, pets, and/or ill family members) and experienced distractions (e.g., household activity and noise) because of the pandemic that, at times, interfered with their ability to fully contribute their presence in the programs. These external stressors paralleled the internal stressors of coping with the pandemic. Personal physical health (e.g., COVID-19 testing/vaccinating, illnesses, need for self-care) and mental health concerns (e.g., depression, anxiety) were among the internal stressors resulting from the pandemic that limited not only student participation but also NICE teacher and YES personnel availability.

On occasion, WISH participants were unable to log into, or lost access to, program sessions due to unstable internet connectivity (Table 2). At times, students who chose to use their cameras had to turn their cameras off to strengthen their connections. Participants who used Zoom, at times, reportedly experienced “Zoom fatigue,” or a sense of energy depletion, lack of ability to focus and/or mental distance from the material presented (Bailenson, 2021). Some students re ported changed sleeping patterns and habits that resulted in tiredness during participation.

#### Disconnection to Places and People.

As discussed, UNMC YES programming did not require students to be on camera to participate in online curriculum activities. Some students chose not to turn on their video during Zoom calls, which is believed to limit students’ sense of community and connection within cohorts (Castelli and Sarvary, 2021). This may have encouraged more overall participation, but it also could have created learning environments that were more disengaged and disconnected. Peer-to-peer connections and mentor-mentee relationships could have been richer if more cameras were enabled to allow participants to see everyone’s facial expressions, read gestures, and observe others’ focused attention.

Without field trip experiences, IResearch club students missed out on making physical connections with people and places associated with their research and real-life learning environments. Overall, participant numbers decreased from 40 middle school students to only 25. During pre-pandemic programming, 92% of participants expressed that they enjoyed field trip experiences. Students expressed that in-person programming, specifically field trips, was an experience that they missed during the 2020–2021 school year (Table 2).

WISH participants did not get the opportunity to sense their place within or “try on” campus life, laboratory environments, or community-based work. In the virtual world, WISH participants did not experience the typical immersive experience with all-day and evening programming to engage students in learning from each other and encourage relationships among peers, graduate/professional students, and faculty mentors. Students lost the opportunity for in-person exposure to campus life, which is a significant motivator to matriculating to college or university (Secore, 2018; Song et al., 2021). Some aspects of programming experienced a drop in quality, such as ability to form new relationships and health profession and research career preparation (Table 2). WISH participant post-program evaluations from 2020 revealed that only half of participants agreed that they met new peers and built new friendships through the WISH program and two-thirds of participants agreed that they developed a mentor through the WISH program. In the pre-pandemic face-to-face setting with WISH students residing on campus, all student participants (100%) reported meeting new people and developing mentor relationships. The inability to have students on-campus with their peers, networking with faculty and staff, and touring facilities which they could attend in the future through health professions education or YES research experiences were likely large factors that impacted the effectiveness of these program components. Through SHPEP-YES WISH, we offered virtual tours and connected students with faculty and staff working on campus in labs and as health professionals, but it appears that these adaptations were not sufficient in supplementing experiences missed in person that contributed to the success of these components.

One significant limitation to having interns participate virtually is the disconnection it creates between mentors and mentees. As one intern stated, “the lack of in-person interaction has also been difficult. While the people directing my project made excellent use of Zoom and remote meeting techniques, I did miss the chance to have in-person meetings.” Not only have the lack of in-person meetings limited connections between people, but they have also disconnected students from place. Not being able to meet or work on-campus limits the connections students have to the campus community and university. As another intern shared, “going to the Public Health department for meetings and collaborations helps build relationships with other interns and network with UNMC faculty.”

The overarching aim of our YES program is to increase the number of NA students in cancer research professions. A key part of developing our workforce in these professions is matriculation into graduate or professional cancer research programs like those on our campus. An important aspect of college recruitment, and thus matriculation and achievement, is campus visitation (Secore, 2018) and engagement with faculty (Carrell and Kurlaender, 2020) on campus. The inability of YES program students to visit campus could negatively influence their decision to pursue a graduate or professional degree program at UNMC.

### Successes.

#### Extended Reach.

The number of student participants in the WISH program grew during the pandemic, increasing from four to six participants when programming shifted to online (Table 2). Students who participated in WISH prior to the pandemic had reported significant barriers to matriculation in college and academic enrichment programs (like YES), including transportation, family commitments to help care for younger siblings, and the need to work to help pay for their essentials. Half of the student participants suggested that the online format reduced or alleviated some of these barriers, eliminating the need to travel and allowing them more flexibility to schedule around family commitments and work.

Having saved funds that would have been spent on lodging and sustenance, we compensated WISH participants with a stipend. This factor could have incentivized participation and contributed to the increase in the number of participants in WISH. An additional potential incentive was the flexible online format. Some students preferred the abbreviated version compared to an all-day, all-week, immersive experience. As one WISH scholar shared, “I think everyone did a great job making the best of an unfortunate situation, especially on short notice. I like the schedule and the format of the online program. I can’t think of anything that I would want to be done differently.”

Prior to the pandemic, the IResearch club programming was limited to only three middle schools that included a NICE program teacher on-site. YES personnel found that the provision of tablets and Microsoft Teams facilitated communication with NICE students at eight schools compared to our three traditional partner schools. This provided us the ability to include students from three additional middle schools, more than doubling our reach. Online platforms, such as Microsoft Teams, also provided an accessible and organized repository of all program materials for students to view and download based on their schedule. This allowed students who were unable to attend live meetings and sessions to be able to view session recordings and related materials on their own time.

Virtual participation allowed UNMC YES personnel and mentors to engage with students outside of the Omaha metropolitan area. For interns, the switch to online media increased the reach of mentors/mentees, allowing interns and mentors in Arizona, Oklahoma, and Nebraska to meet via Zoom biweekly, work together through Microsoft One-Drive, and present their work together in virtual conferences. In fact, one intern in Nebraska, a research participant in Oklahoma, and a mentor in Arizona presented online in a seminar series for the American Indian Cancer Foundation. One intern shared, “the transfer to entirely online due to the COVID-19 meant that I was able to participate in the UNMC YES program, despite the fact that I am living out of state. This actually allowed me to work alongside researchers in Nebraska when, under normal circumstances, I would not have been able to participate at all.”

Analytical features within Canvas will allow us to ascertain which modules within the courses were most utilized by interns, and we will be able to couple this with self-reported data to gain a more complete understanding of each intern’s experience. Examples of what can be monitored include time spent on an online platform, number of login sessions, number of videos accessed and time watching videos, the number of logins and mean length of sessions, timeliness of assignment submissions, and discussion forums posted. The evaluation of Canvas use for interns is currently ongoing.

In addition to participant programming, UNMC YES conducted outreach through an online Research Forum inviting students, staff, faculty, and community to attend. Through the Research Forum, YES faculty and staff presented a program overview and relevant research project findings. Research interns presented their projects through short, live, and pre-recorded poster and PowerPoint presentations. This forum facilitated the engagement of research interns, high school participants, UNMC faculty and staff, NCI personnel, and community to engage in ways not previously considered.

#### Enhanced Sustainability.

The virtual content created during the pandemic will continue to benefit our programming and expand our educational reach even as pandemic restrictions lessen. WISH curricular content and course structure mirrored an online college course, utilizing similar platforms (e.g., Canvas and Zoom) and offering both asynchronous and synchronous lessons. Students’ experiences gave them a taste for what it would be like to participate in an online college course. Many of the high school senior participants who have matriculated to colleges and were registered for online college classes particularly appreciated this opportunity. All the WISH students who participated in the summer of 2020 agreed or strongly agreed that the program provided them with new information about college preparation and helped them prepare for a research education and health professions education program and to advance their career and education goals (Table 2). This data suggests specific outcome successes with the shift to online programming through emergency remote learning.

To respond creatively to the need to provide an alternative curriculum to middle school students participating in the IResearch clubs, we began the process of developing an e-learning module, called “Radon and Cancer,” to support the 2020–2021 school year project. In addition to this, we published a middle school curriculum developed as part of this program on our website (“Cancer Education,” 2020). These are both practices we plan to continue to increase support of partner schools and programs with cancer education curriculum.

In a traditional environment, UNMC YES interns’ schedules would be restricted to the times when mentors are available, which are typically standard workweek hours. Students in school often have schedule conflicts during this time, limiting the number of hours they can participate and engage with mentors in their research. As one COPH intern shared, working remotely “…allows me to work on [tasks at] my most productive times of the day.” Similarly, another intern stated, “had all work been conducted in person, I don’t think I would have been able to maintain working as many hours as I did while keeping up with my classes.” This statement demonstrates that participating online increased participation by providing greater flexibility. This is further supported by hours worked by interns. As seen in Table 2, hours worked doubled from pre-SARS-CoV-2 pandemic compared to during the SARS-CoV-2 pandemic.

The Canvas course created for YES interns participating in the Fred and Pamela Buffett Cancer Center utilizes a combination of videos created by graduate students in cancer research labs, videos created by biomedical corporations, and publicly available content. These videos explain research techniques, including the polymerase chain reaction, western blots, cell culture, and the use of cancer models. An additional module on how to read scientific articles was also made available to students on Canvas. Materials specifically relevant to cancer’s impact on NAs (e.g., articles, seminar links, and the internet sites for organizations such as the American Indian Cancer Foundation) are also incorporated into the Canvas site. We can use this curriculum with future UNMC YES interns and extend our reach by sharing it with other similar programs. This new content has provided a curriculum we would not have had otherwise. This curriculum has been archived and implemented with the middle school IResearch programs, WISH program, and YES research interns.

#### Increased Cultural Relevancy.

Participants of the YES IResearch program reported an increase in learning about NA culture (Table 2) through programming offered during the pandemic (100%) compared to programming offered pre-pandemic. YES IResearch is focused on understanding how exposures to contaminants in the environment can contribute to cancer risk. The previous year’s project on nitrates in surface water was delivered entirely in person and was limited to the focus of local academic partners’ work. Extending our research beyond our region, we were able to introduce students to radon concerns on tribal lands that are not only related to the Omaha metro area but also to AI/AN populations in other urban and rural areas. This helped align our research with Indigenous frameworks that view the natural world in relationship with all beings (Cajete, 2000). IResearch club facilitators hosted regular monthly meetings via Microsoft Teams at times that family and community members could attend with students. This supported the cultural value of acknowledging youth as leaders in the community and creating space for community. Beyond this, meeting virtually allowed the IResearch club to bring in Native guest speakers and Native-specific videos that inspired meaningful dialogue and conversation around culturally relevant topics among students, guests, and teachers.

Similarly, we observed an increase in SHPEP-WISH students reporting that they learned more about NA culture (Table 2), indicating that the YES program’s efficacy in achieving its aim to provide culturally relevant programming was improved through virtual program delivery. This is likely attributed to the program’s ability to invite a wider range of Native guest presenters from across the country to meet with students. Previously, funding and time have restricted the program’s ability to recruit and schedule such presentations. Being able to have guest presenters join us via Zoom accommodated the inclusion of many more AI/AN researchers, health professionals, Indigenous scholars, and cultural educators. Moreover, the use of an online platform allowed us to host sessions with tailored online content designed for NA communities and students from various organizations, such as the American Indian Cancer Foundation. Zoom meetings facilitated space for talking circles, a traditional NA way of passing on knowledge, values, and culture (Wilbur et al., 2001). UNMC YES personnel created a Virtual Talking Circle Guide that was shared with all WISH participants and guest speakers/listeners. This guide detailed protocols developed to allow participants to share thoughts and feelings in a safe context of acceptance and belonging. This included prayer, a topic of focus, a virtual talking stick, and rules for engagement.

Moving to a virtual platform allowed the WISH program to integrate talking circles with out-of-state Indigenous scholars and health care professionals (e.g., a cancer researcher, a veterinary scientist, a nurse) and exceptional guest speakers such as Dr. David Wilson, Director of the National Institutes of Health Tribal Health Research Office (THRO). At the end of the 2020 WISH program, 100% of WISH participants agreed or strongly agreed that they learned more about NA-specific health and wellness (Table 2).

Interns were provided additional culturally relevant content through the use of online learning management systems. The use of Canvas provided an online platform which facilitated learning through videos and corresponding discussion board posts. This content included webinars and videos about cancer research from NA cancer organizations, such as the American Indian Cancer Foundation and Roswell Park Comprehensive Cancer Center’s Center for Indigenous Cancer Research. In addition, students completed culturally relevant e-learning modules developed by the UNMC YES program in partnership with the uBEATS program (“Cancer Education,” 2020).

## DISCUSSION

The abrupt transition to remote delivery of enrichment content and interaction with students presented a threat to our usual ways of establishing rapport, building caring relationships, and fostering engagement with our students through a place-based educational approach. Caring relationships benefit all students but may be especially important to building a sense of belonging for culturally, racially, and ethnically diverse students (Bingham and Okagaki, 2012). Just as in face-to-face interactions, the demeanor of those facilitating the learning climate in a remote setting impacts the tone of the interaction online (Miller, 2021; Chambers et al., 2020). Fortunately, by taking the time to regularly connect with students, we were able to provide encouragement and continue to build relationships and establish rapport with the students in innovative ways that substituted for our regular face-to-face format.

The pandemic made it apparent that many institutions were ill prepared to use technology for educational purposes (Hodges et al., 2020; Trust and Whalen, 2020). As we tried to adapt our practice by using digital means to provide our programs, faculty and staff encountered connectivity issues, difficulties learning how to navigate new virtual platforms, and cybersecurity issues. With a sense of emergency, our goal was to provide temporary access to instruction and educational supports in a way that was quick to set up before returning to a face-to-face format once the emergency subsided. We also continually sought to be aware of and sensitive to students’ and families’ lived experiences. We made sure to reach out in culturally responsive ways to assist with the transition. Not requiring UNMC YES participant use of video cameras for meetings and classes recognizes the stresses of enabling a camera, which among underrepresented minority students includes Zoom fatigue, concerns about appearance, worries about other people being seen behind them, and weak internet connections (Bailenson, 2021; Castelli and Sarvary, 2021). Accessibility to online technology and learning readiness are factors that facilitate a quick shift to emergency remote learning (Hodges et al., 2020). There are numerous hurdles to modifying science programming from in-person to remote delivery that can impede learning, and the situation is made particularly difficult by the educational, financial, and health disparities experienced by AI students (NIEA, 2020; USCCR, 2018).

As many of the YES program activities (i.e., IResearch Club) are still in their pilot phase of implementation, our evaluation data was limited. Moreover, some of our initial curriculum developed was intended to be delivered in person, limiting its effectiveness when utilized remotely. Future program evaluation will include summative assessment of curriculum developed including e-learning modules under development as described above.

The UNMC YES program emphasizes learning through participation in service projects and research to solve local community problems. We employ place-based education immersing students in local heritage, cultures, landscapes, opportunities, and experiences, using these as a foundation for studying cancer. This approach to learning prioritizes the culture, ecology, and history associated with the students’ learning environments (Emekauwa, 2004). Within the UNMC YES program, this educational approach typically involved visiting places (e.g., campuses, rivers, and gardens), and working in multidisciplinary groups including students, community members, and university personnel.

Since it was not safe to ask students to gather in person, leave their homes, or interact with individuals outside their households during the pandemic, the UNMC YES curriculum became entirely dependent on students’ online engagement. Engagement is defined as “devotion of time, energy, value/interest, attitude, learning strategy or even creative thinking in e-learning environments and the motivational and action processes elicited” (Yang et al., 2018). We employed the valuable recommendations made by Chambers et al. (2020) for encouraging students’ online engagement: setting regular touchpoints with students as a wellness and attendance check, tracking assignment completion and submissions, as well as prioritizing wellness, social, and emotional learning.

Despite the many challenges that the pandemic presented, multiple advantages came from how UNMC, NICE, and the community chose to respond. Even though the pandemic prohibited outreach to tribal schools/communities and access to in-person, laboratory-based internships, urban NA youth participation via online platforms increased in some of our programs compared to the previous year. Although intern participants numbers decreased slightly during the pandemic, we observed that hours worked by research interns nearly doubled, demonstrating that offering online research experiences may encourage NA student participation in research pipeline and pathway programs.

In addition, public health undergraduate research internships utilizing remote interviewing and data analysis approaches continued throughout the pandemic. The increases observed could be the result of adapting programming to be offered online. At the middle school level, we reached students beyond our traditional partners due to the accessibility of Microsoft Teams to all students districtwide. High school students expressed that the combination of synchronous and asynchronous teaching increased schedule flexibility and ability to participate and that these were positive factors. Undergraduate public health cancer research interns also appreciated the online format through Zoom as this increased schedule flexibility and included additional culturally responsive material. The challenges we encountered in adapting our programming in response to the SARS-CoV-2 pandemic were varied online accessibility, peripheral stressors, and disconnection to places and peoples. Although these obstacles disrupted our programming, they inspired innovations that have improved the quality of the UNMC YES program by facilitating our cultural relevancy, reach, and sustainability. As one middle school student who was able to participate in the IResearch club because of the changes we made in 2020 noted, “I love this program!”

## Figures and Tables

**Table 1.** Challenges identified at a quarterly advisory board listening session on program delivery during the SARS-CoV-2 Pandemic.

Themes	Theme examples
Varied online accessibility	“We’re treading water. Very few kids are getting online. It’s been a real struggle. They’ve tried to get the kids all Chromebooks and get hot spots out to them. There are some issues of getting internet [to] some of our homes.” (Speaker 6)
	“So, you know, the national average for Internet accessibility in larger cities, is around 80 percent. South Dakota, in rural areas, is talking about rolling out to improve the infrastructure. The governor proposed that. Sixty percent of our students have access to the internet.” (Speaker 2)
	“...you quickly discover when they say they have Internet access at home, that might be a cell phone that has Internet access, but it’s not a laptop. It’s not a robust broadband speed into that.” (Speaker 1)
Peripheral stressors	“I don’t know very few kids online; it’s going to be a mess next year. Even our good students, some of my students, they would go get jobs, do stuff like that, or they’re home taking care of siblings.” (Speaker 6)
	“We have to educationally figure out how to make that a more workable situation and a device for them [students and their families] to use, not only for school but just for life and reaching into the homes and having parents get on the same hot spot and get onto the internet because our society is expecting us to have connections to the internet, social media. That’s how things are functioning.” (Speaker 1)
Disconnection to places and people	“I’m just super thankful that we’ve been in person the entire time. Like, I think that makes a huge difference. And we’re very lucky to have been able to make those relationships from the get-go like you guys who are just now coming back to school. That’s got to be so tough, like you’ve gone eight months without being able to form, like, super strong relationships with your kids.” (Speaker 4)

**Table 2.** Excerpts from evaluation of YES programming.

Program/activity	Pre-SARS-CoV-2 Pandemic^a^	During-SARS-CoV-2 Pandemic^b^
Tribal School Outreach		
Partner school, count	3	0
In-school cancer education participant, count	33	0
YES IResearch Club		
Partner school, count	3	8
Participant, count	40	25
Attendance of all activities	28%	1%
I learned more about cancer from the YES IResearch Club.	50%	100%
I enjoyed the field trip experiences.	92%	-
I was able to make new friends program activities	50%	-
I enjoyed the YES IResearch Club activities on Microsoft Teams	-	100%
I learned more about Native American culture in the YES IResearch Club.	67%	100%
YES WISH		
Participant, count	4	6
Attendance of all activities	90%	100%
I met new peers and built new friendships through the YES WISH program.	100%	50%
The YES WISH program helped me prepare for a research education program.	100%	67%
The YES WISH program helped me prepare for a health professions education program.	100%	67%
Through the YES WISH program, I learned more about Native American culture.	83.3%	100%
YES Research Internship		
Participant, count	8	7
Hours worked, count	817	1,624

a September 1, 2019 to February 29, 2020

b March 1, 2020 through November 30, 2020

## ABBREVIATIONS

AI/AN: American Indian/Alaska Native
NA: Native American
NCI: National Cancer Institute
NICE: Native Indigenous Centered Education
OHKA: Omaha Healthy Kids Alliance
SHPEP: Summer Health Professions Education Program
THRO: Tribal Health Research Office
UNMC: University of Nebraska Medical Center
US: United States
WISH: Weeklong Institute for Students in High School
YES: Youth Enjoy Science
